# *β*-Tocotrienol and *δ*-Tocotrienol as Additional Inhibitors of the Main Protease of Feline Infectious Peritonitis Virus: An In Silico Analysis

**DOI:** 10.3390/vetsci11090424

**Published:** 2024-09-11

**Authors:** Manos C. Vlasiou, Georgios Nikolaou, Kyriakos Spanoudes, Daphne E. Mavrides

**Affiliations:** Department of Veterinary Medicine, University of Nicosia School of Veterinary Medicine, 2414 Nicosia, Cyprus

**Keywords:** FIP, vitamin E, inhibitors, drug discovery

## Abstract

**Simple Summary:**

Feline infectious peritonitis (FIP) is a deadly disease affecting cats, with very few effective treatments available. The disease is caused by a feline coronavirus, and once a cat develops FIP, it is almost always fatal. Researchers are constantly seeking new ways to treat or manage this disease. Vitamin E is known for its strong effects on the immune system, and scientists have now investigated whether certain forms of this vitamin could directly target the virus responsible for FIP. Specifically, different vitamin E analogs, particularly β-tocotrienol and δ-tocotrienol, interact with a key viral enzyme called the 3C-like protease (FIPV-3CLpro), which is essential for the virus’s replication. The study used advanced computer simulations to find that these compounds could potentially inhibit the enzyme, suggesting they might help treat or manage FIP. While this research is still in its early stages, it opens up new possibilities for developing treatments that could improve the prognosis for cats suffering from this devastating disease.

**Abstract:**

Feline infectious peritonitis (FIP) is a severe and invariably fatal disease affecting both domestic and wild felines with limited effective therapeutic options available. By considering the significant immunomodulatory effects of vitamin E observed in both animal and human models under physiological and pathological conditions, we have provided a full in silico investigation of vitamin E and related compounds and their effect on the crystal structure of feline infectious peritonitis virus 3C-like protease (FIPV-3CL^pro^). This work revealed the *β*-tocotrienol and *δ*-tocotrienol analogs as inhibitor candidates for this protein, suggesting their potential as possible drug compounds against FIP or their supplementary use with current medicines against this disease.

## 1. Introduction

Feline coronavirus (FCoV) is a member of the *Coronaviridae* family, a group of enveloped, single-stranded RNA viruses known for their zoonotic potential and capacity to infect a wide range of hosts, including domestic and wild feline species. FCoV is commonly detected in cats, with 50–90% of cats testing positive for FCoV-specific antibodies [1,2]. It is frequently encountered in multi-cat environments like shelters and catteries, where close contact and shared resources facilitate transmission. The virus can cause gastrointestinal infections, mainly in young felines, leading to mild clinical symptoms such as diarrhea, vomiting, mild enteritis, and mild villus atrophy in the small intestine and is referred to as feline enteric coronavirus (FECV) [3,4].

**Figure 1:**
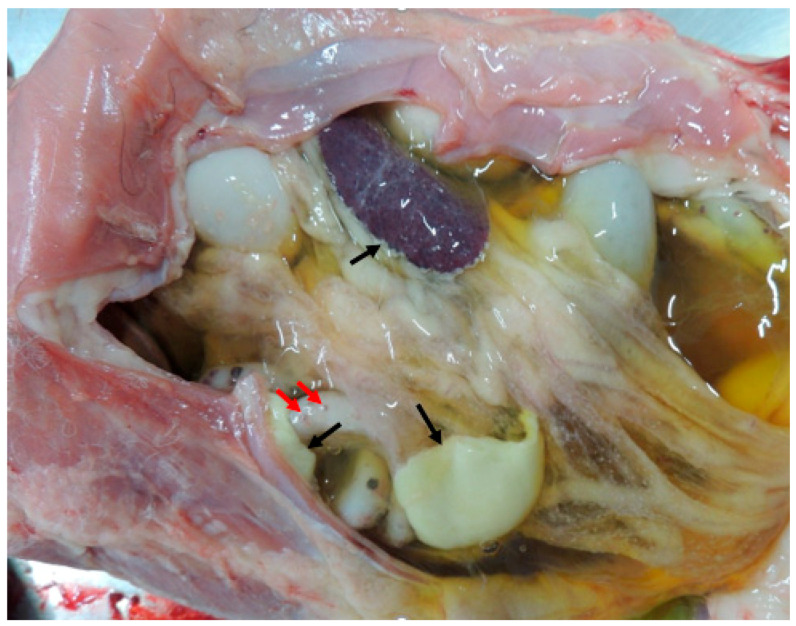
Abdominal cavity in situ, exhibiting a large amount of straw-colored effusion fluid, polymerized fibrin (black arrows), and granulomas on the intestinal serosa (red arrows).

In approximately 5–10% of FCoV-infected cats [5,6], mutations in the virus or recombination events within the host can lead to the development of feline infectious peritonitis (FIP), a complex and devastating disease [7,8]. These genetic changes transform the relatively innocuous virus into a virulent pathogen (referred to as feline infectious peritonitis virus, FIPV) capable of evading the host’s immune system and causing systemic inflammation [9,10]. A broad spectrum of pathological lesions characterizes FIP, determined by interactions between the FIPV and the host’s immune status. Depending on the predominant reaction of the host, the pathological manifestations of FIP are traditionally classified as either the effusive (wet) or non-effusive (dry) form [10,11,12]. In reality, however, most affected individuals will manifest both pathological reactions, even if one predominates over the other (Figure 1). The wet form of FIP is seen in individuals reacting mainly with humoral immunity. This form of the disease is more fulminant, characterized by effusions mainly in the abdominal cavity (peritonitis) and more rarely in the thorax and pericardium [13,14]. Clinically wet forms of FIP are manifested with abdominal swelling, fever, anorexia, and icterus. As humoral immunity appears less appropriate to contain the virus, affected individuals deteriorate rapidly, typically culminating in a fatal outcome.

In contrast, the non-effusive or dry form of the disease is seen in animals reacting primarily with cellular immunity, and it is characterized histologically by granulomas on serous membranes, leptomeninges, and the eyes. Affected animals may show neurological signs, weakness, weight loss, and anorexia. Temporary improvement is occasionally reported in untreated cases of cats with dry forms of FIP; relapse, however, is expected, with the disease occurring in waves [15].

Given the clinical significance of FIP and the potential for FCoV to mutate into FIPV, the study of feline coronavirus is paramount to both veterinary medicine and public health research. So far, there have been no reports of feline coronavirus transmission to humans, but this does not negate the risk of interspecies transmission of the virus. An example of the importance of interspecies transmission cycles can be shown with the recent outbreak of FIP on the island of Cyprus. The virus was reported as a highly pathogenic recombinant between feline coronavirus and canine coronavirus and caused severe illness to spread widely among the feline population. The outbreak was characterized by a 40-fold increase in documented cases compared to the previous years. The diagnosis was confirmed in cats presenting with clinical signs by using RT-qPCR for FCoV on clinical samples (peritoneal, pleural, or cerebrospinal fluid, fine needle aspiration biopsies, or tissue biopsies from granulomatous lesions) [16,17,18]. Strategies to manage and control FCoV infections and ongoing efforts to develop effective vaccines and drugs are therefore crucial for safeguarding feline health and minimizing the risk of transmission to humans in close contact with infected cats.

Computer-aided drug discovery (CADD) holds immense promise in the ongoing battle against feline coronavirus, offering a powerful and innovative approach to identifying potential treatments and antiviral agents. Feline coronavirus, including its severe form, FIP, poses significant challenges to feline health, with limited effective therapeutic options currently available. CADD leverages computational methods, molecular modeling, and simulations to accelerate the drug discovery process [19,20,21]. It allows researchers to screen vast libraries of compounds, predict their binding affinity to viral proteins, and identify potential drug candidates with high specificity, all with reduced time and cost compared to traditional experimental approaches [22,23]. In the context of feline coronavirus, CADD can play a pivotal role in designing novel antiviral drugs by targeting key viral proteins or essential host–virus interactions. By exploring the virus’s molecular structure and interactions at the atomic level, CADD can aid in the rational design of compounds that inhibit viral replication or mitigate the progression of FIP [24,25].

Moreover, CADD can facilitate the repurposing of existing drugs, potentially identifying compounds already approved for other diseases that could be effective against FCoV. This approach expedites drug development and leverages existing safety and pharmacological data, reducing the time required for clinical trials [26,27]. As the field of CADD advances, its potential to contribute significantly to developing effective treatments for FCoV becomes increasingly apparent. By harnessing the computational power of CADD, researchers can accelerate the discovery of antiviral agents, potentially improving the prognosis for cats affected by feline coronavirus infections and offering hope for more successful outcomes in the fight against the challenging disease of FIP.

Herein, we provided a full in silico investigation based on vitamin E and related compounds on their effect on the crystal structure of FIPV-3CL^pro^ against FCoV disease [28]. The 3C-like protease (3CLpro) is a critical enzyme in the lifecycle of many coronaviruses, including feline coronavirus (FCoV). This protease is responsible for processing the viral polyprotein, which is essential for virus replication. Because of its vital role in viral replication, 3CLpro has become a key target for antiviral drug development. This work revealed that the *β*-tocotrienol and *δ*-tocotrienol molecules could act as inhibitors for this protein, suggesting their potential as possible drug compounds against FIP or their supplementary use with current medicines against this disease [29,30].

## 2. Materials and Methods

### 2.1. Computational Tools

In this study, we used all the possible computerized methods that can be performed in computer-aided drug discovery procedures. Virtual screening studies, molecular modeling, molecular docking, molecular dynamics, and density functional theory studies were used in the process. The UCSF CHIMERA molecular modeling system was used for protein visualization and preparation, ChemDraw for ligand design, and AutoDock Vina for molecular docking. Additionally, iGEMDOCK, pharmacological interactions, and virtual screening software were used to evaluate the docking results compared to the inhibitor of the crystal structure of the protease. YASARA software was used for the molecular docking studies, and ORCA (quantum chemistry program) was used for energy minimization of the ligand structures and quantum calculation of the stability and reactivity of the ligand interactions with the amino acid residues. Finally, SwissADME and Swiss Similarity online tools were used to compute physicochemical descriptors and predict ADME parameters, pharmacokinetic properties, drug-like nature, and medicinal chemistry friendliness. All the calculations were performed on workstations with CPU power of Intel Core i9, 32 GB RAM, and 2x NVIDIA GeForce RTX 3060 GPU support.

### 2.2. Computational Methods

Our hypothesis to investigate vitamin E as a potential drug candidate against FCoV infections was based on its antibacterial [31,32,33], antioxidant [34,35,36,37,38], anticancer [39,40], and anti-inflammatory functions. All eight molecules of vitamin E, *α*, *β*, *γ*, *δ*-tocopherol and *α*, *β*, *γ*, *δ*-tocotrienol, were selected for in silico evaluation.

#### 2.2.1. Protein Structure Preparation

The crystal structure of FIPV-3CL^pro^ (PDB: 4ZRO) was retrieved from the protein data bank (www.rcsb.org). Chimera software [41] was utilized to prepare the protein structure. All water molecules were deleted, and the required chain was kept. Heteroatoms excluding the co-crystallized ligand were removed and polar hydrogens were added to the crystal structure. The receptor and the co-crystal ligand were selected to define the binding site and the XYZ coordinates were noted down for further use. The co-crystal ligand was then removed and the protein structure was stored in PDB format for further use.

#### 2.2.2. Database Preparation

We used the ZINC database for the virtual screening process, using the filters (biological active molecules, commercially available). Before we downloaded the structures from the database, we compressed them in SDF format. The Schrodinger suit’s LigPrep [42] module filtered and prepared the downloaded database based on their ring size to obtain 400 molecules.

#### 2.2.3. Structure-Based Virtual Screening

PyRx is a virtual screening platform that can be used in computer-aided drug discovery to screen libraries of compounds against potential targets. PuRx 0.8 [43] software (https://pyrx.sourceforge.io) is an open-source tool for virtual screening which has integrated Autodock Vina [44], Python shell, and Open Babel [45] on a single platform. The ligands were handled in SDF format, and the energy was minimized. The SDF files were converted into pdbqt format. The prepared protein structure was formatted further into PDBQT format alongside the ligands to be used in AUTODOCK. The grid box was configured with dimensions of X = 25.00, Y = 45.00, and Z = 30.00 Å. All ligands were docked against the 4ZRO protein using AutoDock Vina. For evaluation purposes, the bound inhibitor N-(tert-butoxycarbonyl)-L-seryl-L-valyl-N-{(2S)-5-ethoxy-5-oxo-1-[(3S)-2-oxopyrrolidin-3-yl] pentan-2-yl}-L-leucinamide (the co-crystal ligand of the 4ZRO protein) was prepared similarly and docked against the protein structure. Based on the binding affinity results from Vina docking, the top 10 molecules were selected for further evaluation. Structural analysis and visualization of the interactions between the ligands and active site amino acid residues were performed using Chimera software.

#### 2.2.4. Molecular Docking by iGEMDOCK

iGEMDOCK is a graphical tool designed for identifying pharmacological interactions and conducting virtual screening. Experimental results demonstrate that iGEMDOCK achieves a 78% success rate, with root-mean-square deviations (RMSDs) under 2.0 angstroms across 305 protein–compound complexes. The crystal structure of the 4ZRO protein underwent energy minimization using the AMBER 96 force field. The top 10 scoring ligands from the virtual screening, along with the co-crystal ligand, were prepared using the ORCA software. ORCA is a quantum chemistry program that includes several electronic structure methods [46]. Using the Hartee Fork theory [47], we were able to carry out geometry optimizations of the candidate ligands. The following scoring function is used to reduce the number of false positives:

*E*_*tot*_ = *E*_*bind*_ + *E*_*pharma*_ + *E*_*ligpre*_

where *E_bind_* is the empirical binding energy used during the molecular docking; *E_pharma_* is the energy of binding-site pharmacophores; *E_ligpre_* is a penalty value if the ligand does not satisfy the ligand preferences. *E_pharma_* and *E_ligpre_* were used to improve the number of true positives by discriminating active compounds. The empirical binding energy (*E_bind_*) is given as follows:

*E_bind_* = *E_inter_* + *E_intra_ + E_penal_*

where *E_inter_* and *E_intra_* are the intermolecular and intramolecular energy, respectively, and *E_penal_* is a large penalty value if the ligand is out of range of the search box. In this study, *E_penal_* was set to 1000.

#### 2.2.5. Binding Free Energy Estimation

After performing iGEMDOCK docking, a Maestro pose viewer file was generated, which contained detailed information about the docked complexes of all ligands. This pose viewer file was then used as input in the Prime [48] module of Schrodinger [49,50] to estimate the free binding energy of the docked complexes. The overall binding energy (ΔGbind) was calculated using the equation ΔGbind = EMM + Gsolv − ΤΔS, where EMM represents the molecular mechanics energy (gas phase), Gsolv is the solvation free energy (gas phase), and ΤΔS accounts for the conformational entropy changes during complex formation.

#### 2.2.6. In Silico ADMET Studies

ADMET, which stands for absorption, distribution, metabolism, elimination, and toxicity, is a vital profile used to prevent failures in the later stages of clinical trials. The top ten compounds identified through virtual screening were assessed for their ADME properties using the Swiss ADME web server (https://www.swissadme.ch/). The server used the SMILES (Simplified Molecular Input Line Entry System) of each compound to predict different ADME parameters, such as oral absorption, bioavailability score, and inhibition of different cytochrome P enzymes. Topological Polar Surface Area (TPSA) indicates a molecule’s polarity, which affects its absorption in the gastrointestinal tract and its ability to cross the blood–brain barrier. The iLOGP tool estimates the octanol–water partition coefficient based on the free energy of solvation using the GB/SA (Generalized-Born and solvent-accessible surface area) model. ESOL LogS predicts the compound’s aqueous solubility. Cytochrome P450 enzymes are essential in the metabolism and elimination of drug metabolites, and inhibiting one or more of these enzymes can result in drug–drug interactions, drug accumulation, and adverse effects. The PAINS (Pan Assay Interference Compounds) alert system is used to identify potential false positives or promiscuous compounds in drug discovery.

OSIRIS [51] property explorer (https://www.organic-chemistry.org/prog/peo/, accessed on 7 July 2024) was used to study the top 4 hits of tumorigenicity, mutagenicity, irritant, and reproductive risks. The SMILES of the compounds were used as an input for the OSIRIS portal, which returned the toxicity results in color codes (red for high risk, orange for medium, and green for low risk).

### 2.3. Molecular Dynamics (MD) Studies

The YASARA software environment [52] is a molecular modeling and molecular dynamics tool that supports automatic simulation set-up and provides the robust calculation of free energies. Molecular dynamics studies were conducted using YASARA software, employing an explicit solvent environment with periodic boundary conditions and an orthorhombic simulation box (10 × 10 × 10 Å). The system was solvated with explicit water molecules and neutralized by adding Na^+^ and Cl^−^ ions of 0.15 M. The entire simulation was performed using the AMBER96 force field. Before the production run, the system underwent a standard equilibration protocol to minimize and pre-equilibrate it. A 100 ns production simulation was then carried out under normal pressure and temperature (NPT) conditions at a pressure of 1.1325 bar and a temperature of 300 K. The system’s coordinates and energy were saved in the trajectory every 10 ps. A simulation interaction diagram was utilized to analyze the stability of the complex.

### 2.4. Density Functional Theory (DFT) Studies

Density functional theory studies on B3LYP/6 311++G (d, p) were performed to discriminate the chemical reactivity between vitamin E derivatives. In addition, we were able to calculate the molecular orbitals of the molecules. The value of the energy difference between HOMO and LUMO and the highest occupied molecular orbital (EHOMO) and lowest unoccupied molecular orbital (ELUMO) energies play a significant role in the stability and reactivity of molecules [53,54]. The EHOMO energies of molecules show the molecule’s ability to donate electrons. On the other hand, ELUMO characterizes the ability of the compound to accept electrons. Electronegativity (χ) measures an atom’s power to attract a bonding pair of electrons. Based on the equation χ = −(EHOMO + ELUMO)/2, a larger Δgap always indicates lower chemical reactivity and higher kinetic stability of the investigated species [55,56]. The simultaneous effect of different parameters causes the chemical reactivity of molecules. The distribution and energy of HOMO are important parameters to explain the antioxidant potential of phenolic antioxidants. The electron-donating capacity of the molecule can be predicted by looking at the energy values of HOMO [57].

## 3. Results

**Figure 2:**
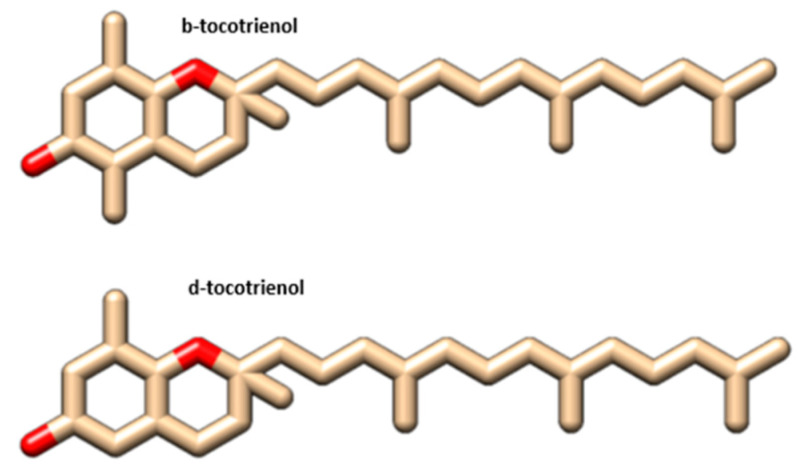
*β* and *δ*-tocotrienol structures.

**Figure 3:**
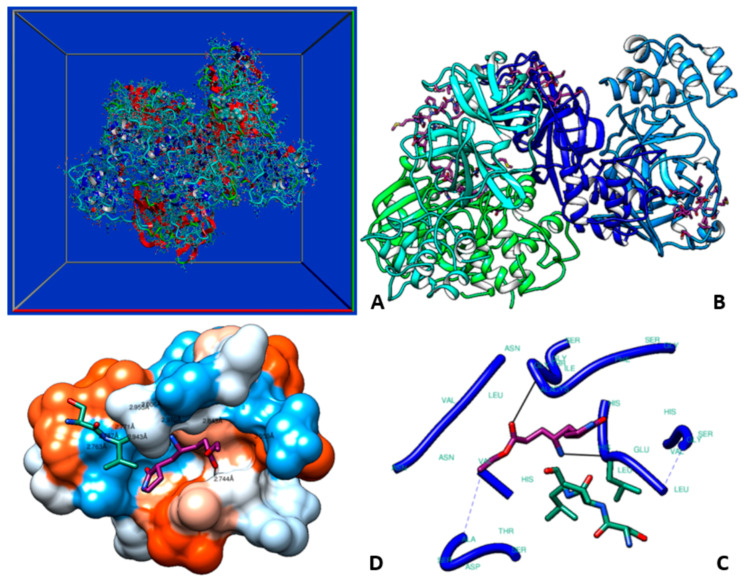
(**A**) Simulation box of the molecular dynamics studies. (**B**) Binding sites of *β* and *δ*-tocotrienol. (**C**) Amino acid residue interactions of the two lead candidates. *β* (green color) and *δ*-tocotrienol (purple color). (**D**) Binding pocket of β (green color) and δ-tocotrienol (purple color).

**Figure 4:**
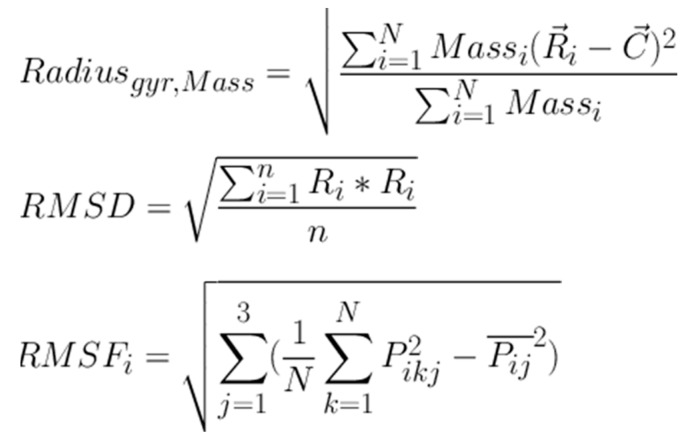
Equations used in radius mass calculations and RMSD and RMSF value calculations.

**Figure 5:**
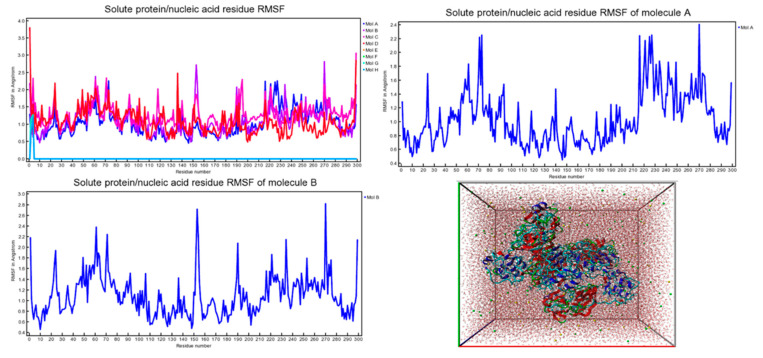
RMSF values through the amino acid residues interactions (Molecule A: *β*-tocotrienol, Molecule B: *δ*-tocotrienol). Root-mean-square fluctuation (RMSF) is a metric commonly used in molecular dynamics (MD) simulations to quantify the flexibility of specific atoms or residues in a molecule, typically a protein.

Molecular docking analysis details the interaction energies and specific amino acid residues involved in both *van der* Waals and hydrogen bond interactions for the complexes formed between *β*-tocotrienol and *δ*-tocotrienol with the FIPV-3CL^pro^ protein. The structures of *β*-tocotrienol and *δ*-tocotrienol molecules can be seen in Figure 2. The energy results of the amino acid residue interactions with the two candidates on the protein can be found in Table 1. Figure 3 illustrates the simulation box of the molecular dynamics’ studies. Additionally, we can see the binding sites of *β* and *δ*-tocotrienol, as well as amino acid residue interactions of the two lead candidates of *β* (green color) and *δ*-tocotrienol (purple color). The binding pockets of *β* (green color) and *δ*-tocotrienol (purple color) are also depicted in Figure 4. Figure 4 depicts the equations used for the calculations of radius mass, RMSD, and RMSF values. Figure 5 includes the whole simulation process for the RMSF values. The remaining work is listed in the Supplementary Materials.

**Figure 6:**
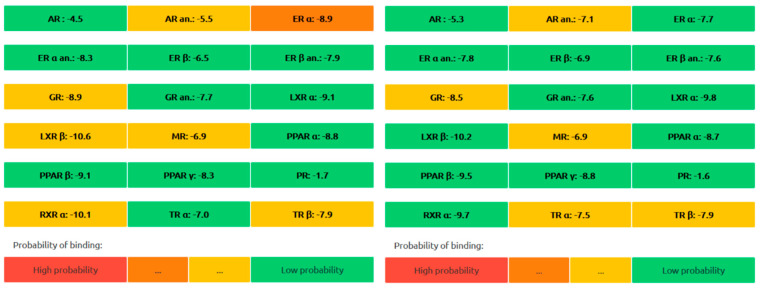

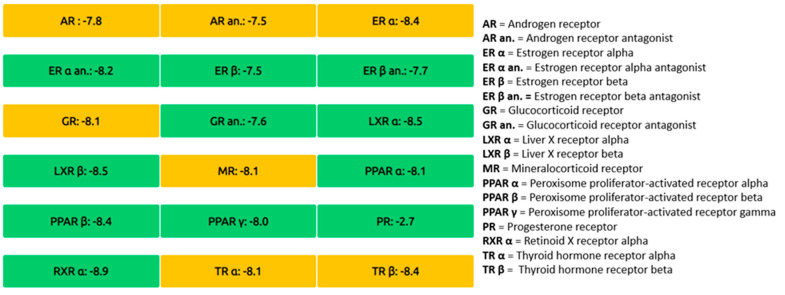
Endocrine disruption potential of *β*-tocotrienol (**left**) and *δ*-tocotrienol (**right**) obtained from the Endocrine Disruptome. Orange and yellow coloring indicates medium probability of binding, while green coloring indicates low probability of binding.

The comparative analysis, which outlines the chemical properties, pharmacokinetics, and toxicity profiles of *β*-tocotrienol and *δ*-tocotrienol, as well as highlighting similarities and differences between the two compounds, is shown in Table 2. The endocrine disruption potential of *β*-tocotrienol (left) and *δ*-tocotrienol (rights) obtained from the Endocrine Disruptome is found in Figure 6. The orange and yellow coloring indicates a medium probability of binding, while the green coloring indicates a low probability of binding. Both candidate substances show a low probability of binding to the receptors, thus indicating limited toxicity.

**Figure 7:**
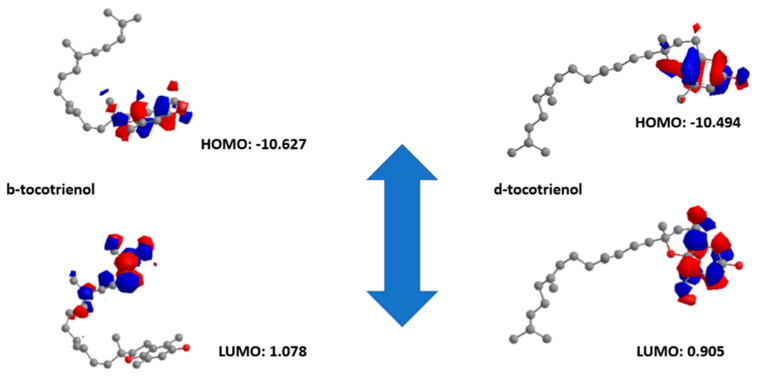
HOMO and LUMO energies of *β* and *δ*-tocotrienol molecules.

The first candidate has a LUMO orbital equal to 1.078 eV, while the HOMO orbital equals −10.627 eV (Egap −9.549). On the other hand, regarding the second molecule, the LUMO orbital equals 0.905 eV, and the HOMO orbital equals −10.494 eV (Egap −10.044). Based on that, δ-tocotrienol is more electronegative than β-tocotrienol (larger Egap). The quantum chemical descriptors of the molecules can be found in Figure 7. The results show that δ-tocotrienol has higher reactivity based on the calculated energy gap of the HOMO and LUMO orbitals. These results agree with the docking work.

## 4. Discussion

### 4.1. Virtual Screening and Molecular Docking

The initial virtual screening using PyRx and AutoDock Vina identified several vitamin E derivatives with promising binding affinities to the FIPV-3CL^pro^ (PDB: 4ZRO) protein. Among these, *β*-tocotrienol and *δ*-tocotrienol exhibited the highest binding affinities, with docking scores suggesting strong interactions with the active site of the protease. Specifically, the docking scores indicated that these compounds could form stable interactions within the active site, potentially inhibiting the protease activity crucial for FIPV replication. The top ten scoring ligands from the virtual screening were further refined using iGEMDOCK, which offers more detailed insights into the binding interactions. This additional docking study confirmed the high binding affinities of *β*-tocotrienol and *δ*-tocotrienol, demonstrating significant interactions with key residues in the FIPV-3CL^pro^ active site. The empirical binding energies (*E_bind_*) calculated by iGEMDOCK further supported these findings, with *β*-tocotrienol and *δ*-tocotrienol exhibiting lower *E_bind_* values, indicating stronger binding.

Further binding free energy (ΔG_bind_) estimations using the Prime module provided additional insights into the stability of the ligand–protein complexes. Both *β*-tocotrienol and *δ*-tocotrienol exhibited favorable ΔG_bind_ values, reinforcing their potential as strong inhibitors of FIPV-3CL^pro^. The consistent results across different docking tools underscore the reliability of these findings, suggesting that *β*-tocotrienol and *δ*-tocotrienol could be potent antiviral agents against FIPV.

### 4.2. ADMET and Molecular Dynamics (MD) Studies

The ADMET profiles of the top ten compounds were assessed to ensure drug-likeness and safety. *β*-tocotrienol and *δ*-tocotrienol showed favorable ADME properties, including good oral absorption, high bioavailability, and minimal inhibition of cytochrome-P450 enzymes, reducing the risk of drug–drug interactions. These properties are crucial for the development of any potential therapeutic agent, as they determine the compound’s effectiveness and safety in a biological system. The OSIRIS property explorer indicated low risks of tumorigenicity, mutagenicity, irritancy, and reproductive toxicity for these compounds, further validating their potential as safe drug candidates.

MD simulations performed using YASARA demonstrated the stability of the *β*-tocotrienol and *δ*-tocotrienol complexes with FIPV-3CL^pro^ over a 100 ns period. The root-mean-square deviation (RMSD) and root-mean-square fluctuation (RMSF) analyses confirmed that both ligand–protein complexes maintained stable interactions throughout the simulation, with minimal deviations indicating strong and stable binding. The low RMSD and RMSF values observed suggest that these compounds are not only strong binders but also form stable complexes over time, which is essential for effective inhibition.

### 4.3. Comprehensive Analysis and Future Directions

The computational studies presented herein provide a comprehensive analysis of vitamin E derivatives as potential inhibitors of FIPV-3CL^pro^. *β*-tocotrienol and *δ*-tocotrienol emerged as the most promising candidates based on multiple criteria, including binding affinity, stability, and ADMET profiles. The strong interactions observed in molecular docking studies were corroborated by binding free energy estimations and MD simulations, highlighting the stability of these complexes. The favorable ADMET profiles suggest that *β*-tocotrienol and *δ*-tocotrienol could be developed into safe and effective drugs for FIP treatment. Additionally, their antioxidant properties could provide synergistic benefits when used in combination with existing therapies, enhancing the overall treatment efficacy.

The DFT (density functional theory) analysis further supported the potential of *β*-tocotrienol and *δ*-tocotrienol, demonstrating their high reactivity and electron-donating capabilities, which are crucial for the effective inhibition of viral proteases. This quantum mechanical analysis provided additional validation of the electronic properties that contribute to the binding efficacy observed in docking studies. The combination of DFT results with empirical binding energies and MD simulation data creates a robust framework for understanding the molecular mechanisms underlying the inhibition of FIPV-3CL^pro^ by these compounds. Preliminary work that we have performed on the nucleoside analog GS-441524, which is used for FIP, indicated less molecular reactivity than our candidate molecules based on density functional theory studies and less binding affinity based on docking studies. This gave us hope that the new studied candidates might be more efficient in a field trial.

Cell membranes are primarily composed of lipid bilayers, which are hydrophobic (water-repelling) in nature. Lipophilic drugs or molecules can more easily diffuse through these lipid bilayers compared to hydrophilic (water-soluble) drugs. Because of their ability to pass through cell membranes, lipophilic drugs often have better absorption rates in the gastrointestinal tract when taken orally. This can increase the drug’s bioavailability. Lipophilic drugs tend to bind more effectively to plasma proteins, such as albumin. This binding can protect the drug from being rapidly metabolized or excreted, leading to a longer duration of action. Lipophilic drugs can be stored in adipose (fat) tissue, allowing for a slower release into the bloodstream, which can prolong the drug’s effects. In some cases, lipophilic drugs can be used in combination with other lipophilic compounds to produce a synergistic effect, enhancing the overall therapeutic outcome. So, based on the vitamin E analog’s lipophilicity and the fact that vitamin E components can be administered as supplements with no adverse effects, we suggest that *β* and *δ* tocotrienols can be used in field trials both as drug candidates or enhancers to an existing therapy.

This study identified *β*-tocotrienol and *δ*-tocotrienol as potent inhibitors of FIPV-3CL^pro^, providing a strong basis for further experimental validation and development as therapeutic agents against FIP. The integration of various computational methods allowed for a thorough evaluation of these compounds, paving the way for new treatment strategies to combat this fatal feline disease. Future research should focus on the in vitro and in vivo validation of these findings, as well as exploring potential formulation and delivery methods to maximize the therapeutic efficacy of *β*-tocotrienol and *δ*-tocotrienol in clinical settings.

## 5. Conclusions

This study provides a comprehensive computational analysis of vitamin E analogs, specifically *β*-tocotrienol and *δ*-tocotrienol, as potential inhibitors of the feline infectious peritonitis virus (FIPV) main protease. Our molecular dynamics simulations have demonstrated that these compounds exhibit strong and stable binding affinities to the protease active site, suggesting their potential efficacy in disrupting viral replication. The strong interaction profiles of *β*-tocotrienol and *δ*-tocotrienol with the FIPV main protease, as revealed by our in silico studies, make them promising candidates for further in vitro and in vivo investigations. These findings open up new avenues for the development of novel therapeutic strategies against FIP, a disease with limited effective treatments. The study highlights the potential for using vitamin E analogs in combination with established antiviral therapies, such as remdesivir. The complementary mechanisms of action suggest that such a combination could potentiate the therapeutic effects, reduce the likelihood of drug resistance, and improve overall clinical outcomes in feline patients suffering from FIP. The synergistic use of vitamin E with other antiviral agents represents a promising strategy that warrants further exploration in clinical settings. This work not only identifies *β*-tocotrienol and *δ*-tocotrienol as strong candidates for FIPV protease inhibition but also suggests a broader role for vitamin E analogs in enhancing antiviral therapy. Future studies should focus on validating these computational predictions through experimental assays and clinical trials to fully realize the therapeutic potential of these compounds in combating FIP.

## Figures and Tables

**Table 1:** Energies and amino acid residues of the two most promising candidates (*β*-tocotrienol and *δ*-tocotrienol) on the FIPV-3CL^pro^ protein.

Complex	Total Energy (KJ/mole)	Energy VDW (KJ/mole)	Energy H_bond_ (KJ/mole)	Amino Acid Residue H_bonds_	Amino Acid Residue VDW Interactions
*β*-tocotrienol-FIPV-3CLpro	−94.92	−91.42	−3.5	PHE ^111^	MET ^6^, GLN ^8^, GLU ^291^, ARG ^294^
*δ*-tocotrienol-FIPV-3CLpro	−98.23	−86.32	−11.9	PHE ^111^, VAL ^127^	GLY ^122^, GLN ^8^, PHE ^111^, ASN ^112^, GLU ^291^, ARG ^294^, VAL ^299^

**Table 2:** ADMET studies for *β* and *δ* tocotrienol.

Formula	C_28_H_42_O_2_	C_27_H_40_O_2_	Formula	C_28_H_42_O_2_	C_27_H_40_O_2_
Molecular weight	410.63 g/mol	396.61 g/mol	Total clearance	0.814 log mL/min/kg	0.847 log ml/min/kg
Num. of heavy atoms	30	29	Renal OCT2 substrate	No	No
Num. of aromatic heavy atoms	6	6	AMES toxicity	No	No
Num. of rotatable bonds	9	9	Max. tolerated dose	0.45 log mg/kg/day	0.729 log mg/kg/day
Num. of H-bond acceptors	2	2	hERG I Inhibitor	No	No
Num. of H-bond donors	1	1	hERG II Inhibitor	Yes	Yes
Molar refractivity	132.88	127.2	Oral rat acute toxicity (LD50)	2.18 mol/kg	1.945 mol/kg
Log P_o/w_	5.14	5.35	Oral rat chronic toxicity (LOAEL)	2.967 log mg/kg_bw/day	2.956 log mg/kg_bw/day
Log S	−7.57	−7.26	Hepatotoxicity	No	No
GI absorption	Low	Low	Skin sensitisation	No	No
BBB permeant	No	No	*T. pyriformis* toxicity	1.127 log ug/L	1.182 log ug/L
P-gp substrate	Yes	Yes	Minnow toxicity	−2.5 mM	−4.247 mM
CYP1A2 inhibitor	No	No		No	No
CYP2C19 inhibitor	No	No			
CYP2C9 inhibitor	No	No			
CYP2D6 inhibitor	No	No			
CYP3A4 inhibitor	No	Yes			
Log K_p_ (skin permeation)	−2.46 cm/s	−2.63 cm/s			
Lipinski	Yes, 1 violation: MLOGP > 4.15	Yes, 1 violation: MLOGP > 4.15			
Bioavailability Score	0.55	0.55			

## Data Availability

The original contributions presented in the study are included in the article/Supplementary Materials, further inquiries can be directed to the corresponding author.

## Supplementary Materials

The following are available online at https://www.mdpi.com/article/10.3390/vetsci11090424/s1: Figure S1: ADME results of *β* and *δ*-tocotrienol molecules, Figure S2: Molecular properties calculated for *β*-tocotrienol, Figure S3: Calculated toxicity results for *β*-tocotrienol, Figure S4: Molecular properties calculated for *δ*-tocotrienol, Figure S5: Calculated toxicity results for *δ*-tocotrienol.
