# Supplementary material for: β-Tocotrienol and δ-Tocotrienol as Additional Inhibitors of the Main Protease of Feline Infectious Peritonitis Virus: An In Silico Analysis
Source: Vet Sci. 2024 Sep 11;11(9):424. doi: 10.3390/vetsci11090424 (PMC11435718; doi:10.3390/vetsci11090424)
Supplement: Supplementary file 1 [file vetsci-11-00424-s001.zip › vetsci-3116485-supplementary.pdf]

Supplementary Material

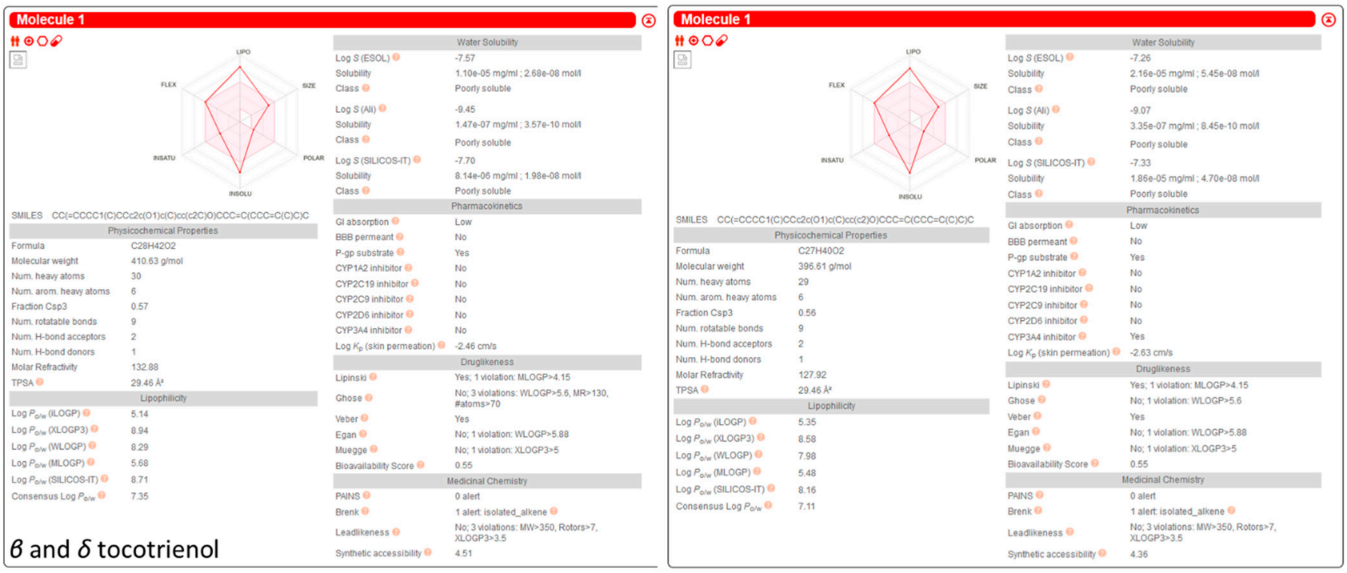

Figure S1. ADME results of *β* and *δ*-tocotrienol molecules.

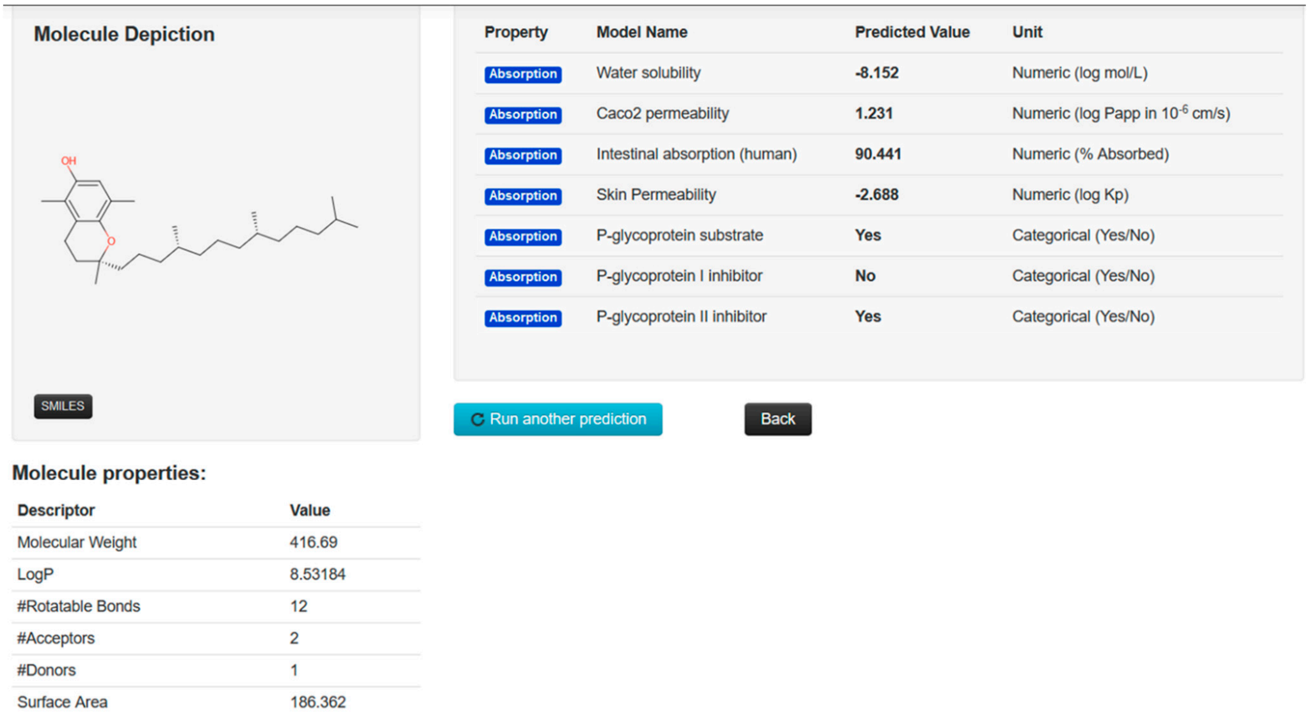

Figure S2. Molecular properties calculated for *β*-tocotrienol.

Property	Model Name	Predicted Value	Unit
Distribution	VDss (human)	**0.747**	Numeric (log L/kg)
Distribution	Fraction unbound (human)	**0**	Numeric (Fu)
Distribution	BBB permeability	**0.918**	Numeric (log BB)
Distribution	CNS permeability	**-1.454**	Numeric (log PS)

Property	Model Name	Predicted Value	Unit
Metabolism	CYP2D6 substrate	**No**	Categorical (Yes/No)
Metabolism	CYP3A4 substrate	**Yes**	Categorical (Yes/No)
Metabolism	CYP1A2 inhibitior	**No**	Categorical (Yes/No)
Metabolism	CYP2C19 inhibitior	**No**	Categorical (Yes/No)
Metabolism	CYP2C9 inhibitior	**No**	Categorical (Yes/No)
Metabolism	CYP2D6 inhibitior	**No**	Categorical (Yes/No)
Metabolism	CYP3A4 inhibitior	**No**	Categorical (Yes/No)

Property	Model Name	Predicted Value	Unit
Metabolism	CYP2D6 substrate	**No**	Categorical (Yes/No)
Metabolism	CYP3A4 substrate	**Yes**	Categorical (Yes/No)
Metabolism	CYP1A2 inhibitior	**No**	Categorical (Yes/No)
Metabolism	CYP2C19 inhibitior	**No**	Categorical (Yes/No)
Metabolism	CYP2C9 inhibitior	**No**	Categorical (Yes/No)
Metabolism	CYP2D6 inhibitior	**No**	Categorical (Yes/No)
Metabolism	CYP3A4 inhibitior	**No**	Categorical (Yes/No)

Property	Model Name	Predicted Value	Unit
Excretion	Total Clearance	**0.814**	Numeric (log ml/min/kg)
Excretion	Renal OCT2 substrate	**No**	Categorical (Yes/No)

Property	Model Name	Predicted Value	Unit
Toxicity	AMES toxicity	No	Categorical (Yes/No)
Toxicity	Max. tolerated dose (human)	0.45	Numeric (log mg/kg/day)
Toxicity	hERG I inhibitor	No	Categorical (Yes/No)
Toxicity	hERG II inhibitor	Yes	Categorical (Yes/No)
Toxicity	Oral Rat Acute Toxicity (LD50)	2.18	Numeric (mol/kg)
Toxicity	Oral Rat Chronic Toxicity (LOAEL)	2.967	Numeric (log mg/kg_bw/day)
Toxicity	Hepatotoxicity	No	Categorical (Yes/No)
Toxicity	Skin Sensitisation	No	Categorical (Yes/No)
Toxicity	*T.Pyriformis* toxicity	1.127	Numeric (log ug/L)
Toxicity	Minnow toxicity	-2.5	Numeric (log mM)

Figure S3. Calculated toxicity results for *β*-tocotrienol.

Molecule Depiction

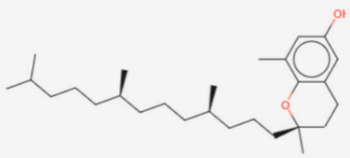

SMILES

Molecule properties:

Descriptor	Value
Molecular Weight	402.663
LogP	8.22342
#Rotatable Bonds	12
#Acceptors	2
#Donors	1
Surface Area	179.997

Property	Model Name	Predicted Value	Unit
Absorption	Water solubility	-8.047	Numeric (log mol/L)
Absorption	Caco2 permeability	1.284	Numeric (log Papp in 10^{-6} cm/s)
Absorption	Intestinal absorption (human)	91.075	Numeric (% Absorbed)
Absorption	Skin Permeability	-2.667	Numeric (log Kp)
Absorption	P-glycoprotein substrate	No	Categorical (Yes/No)
Absorption	P-glycoprotein I inhibitor	Yes	Categorical (Yes/No)
Absorption	P-glycoprotein II inhibitor	Yes	Categorical (Yes/No)
Distribution	VDss (human)	0.925	Numeric (log L/kg)
Distribution	Fraction unbound (human)	0	Numeric (Fu)
Distribution	BBB permeability	0.727	Numeric (log BB)
Distribution	CNS permeability	-1.604	Numeric (log PS)
Metabolism	CYP2D6 substrate	No	Categorical (Yes/No)
Metabolism	CYP3A4 substrate	Yes	Categorical (Yes/No)
Metabolism	CYP1A2 inhibitior	No	Categorical (Yes/No)
Metabolism	CYP2C19 inhibitior	No	Categorical (Yes/No)
Metabolism	CYP2C9 inhibitior	No	Categorical (Yes/No)
Metabolism	CYP2D6 inhibitior	No	Categorical (Yes/No)
Metabolism	CYP3A4 inhibitior	No	Categorical (Yes/No)

Figure S4. Molecular properties calculated for *δ*-tocotrienol.

Excretion	Total Clearance	**0.847**	Numeric (log ml/min/kg)
Excretion	Renal OCT2 substrate	**No**	Categorical (Yes/No)
Toxicity	AMES toxicity	**No**	Categorical (Yes/No)
Toxicity	Max. tolerated dose (human)	**0.729**	Numeric (log mg/kg/day)
Toxicity	hERG I inhibitor	**No**	Categorical (Yes/No)
Toxicity	hERG II inhibitor	**Yes**	Categorical (Yes/No)
Toxicity	Oral Rat Acute Toxicity (LD50)	**1.945**	Numeric (mol/kg)
Toxicity	Oral Rat Chronic Toxicity (LOAEL)	**2.956**	Numeric (log mg/kg_bw/day)
Toxicity	Hepatotoxicity	**No**	Categorical (Yes/No)
Toxicity	Skin Sensitisation	**No**	Categorical (Yes/No)
Toxicity	*T.Pyriformis* toxicity	**1.182**	Numeric (log ug/L)
Toxicity	Minnow toxicity	**-4.247**	Numeric (log mM)

Figure S5. Calculated toxicity results for δ-tocotrienol.
